# Clinical efficacy of 0.01% atropine in retarding the progression of myopia in children

**DOI:** 10.1007/s10792-020-01658-0

**Published:** 2020-11-17

**Authors:** Qi Zhao, Qian Hao

**Affiliations:** Department of Ophthalmology, The Second Hospital of Dalian Medical University, 467 Zhongshan Road, Shahekou District, Dalian, People’s Republic of China

**Keywords:** 0.01% atropine, Myopia progression, Children

## Abstract

**Purpose:**

To investigate the clinical efficacy of 0.01% atropine in slowing the progression of myopia in children and to evaluate the influence of 0.01% atropine on secretion of basal tear and stability of tear film.

**Methods:**

Eighty children aged 5–14 years with myopia, 40 were randomly divided into two groups consisting of those who received spectacles in addition to 0.01% atropine (SA group) and those who received only spectacles (S group). The remaining 40 children who were wearing orthokeratology (OK) lenses for 3 months were randomly divided into two groups comprising those who received OK lenses in addition to 0.01% atropine (OKA group) and those who received only OK lenses (OK group). Comprehensive ophthalmologic examinations, including slit-lamp examination, visual acuity testing, autorefraction, intraocular pressure, axial length (AL), corneal topography, Schirmer’s test, and tear film break-up time (TBuT), were performed before treatment and after every 3 months treatment.

**Results:**

During the follow-up visits, evidently better spherical equivalent (SE) control over 3, 6 and 12 months was observed in the SA and OKA groups compared with the S and OK groups. The AL over 3, 6, and 12 months was evidently inhibited in the SA and OKA groups compared with the S and OK groups. No statistically significant differences in Schirmer’s test and TBuT results were observed between the S and SA groups and between the OK and OKA groups. However, statistically significant differences were found in TBuT results between before treatment and after 3 months treatment in the OK group (*P* < 0.05, paired *t* test) and the OKA group (*P* < 0.05, paired *t* test).

**Conclusions:**

0.01% atropine can effectively control myopia progression and axial elongation regardless of combined treatment with spectacles or OK lenses. And 0.01% atropine has no evident effect on Schirmer’s test and TBuT results; however, researchers also found that Schirmer’s test and TBuT results showed a tendency to reduce after treatment with 0.01% atropine.

## Introduction

Myopia is a refractive error in which the refractive power is too large relative to the axial length of the eye. That is to say, in the static state of adjustment, external parallel light enters the eye and focuses before the photoreceptor layer of the retina, resulting in blurred far vision and clear near vision. Once myopia is formed, it will be irreversible. Nowadays, myopia is a common refractive error in children [1]. The prevalence of pediatric myopia reached to very high levels worldwide, especially in East Asian countries such as China [2]. Researchers predicted that approximately 50% of the population will be myopic by 2050, almost 1 billion people will exhibit high myopia [3]. As myopia tends to progress more rapidly when it occurs in a younger age and myopia progresses until adulthood [4, 5], controlling the development of myopia in adolescents is an urgent problem. A high myopia of ≥ 6 D increases the risk for vitreous opacity, macular degeneration, posterior sclera staphyloma, choroidal neovascularization, which is closely related to cataract, glaucoma, retinal detachment, and even blindness [2]. Multiple interventional methods, including pharmaceutical and optical treatments, were proposed to slow down myopia progression in children [6]. The muscarinic receptor antagonist 0.01% atropine was considered to be the most effective and safe treatment [6, 7]. However, we still require much experimental data to evaluate the clinical efficacy of 0.01% atropine in controlling myopia and providing guidance for clinical practice. In addition, concerns exist regarding whether accumulation of drug toxicity occurs and whether long-term use of 0.01% atropine would cause dry eyes. Therefore, analyzing the influence of 0.01% atropine on tear secretion and stability of tear film is also necessary.

## Materials and methods

### Patients

We included 80 children of ages 5–14 years with myopia at the Second Affiliated Hospital of Dalian Medical University during the years January 2019–April 2020. Before the experiment, the investigators explained the expected benefits and potential risks of using 0.01% atropine and OK lenses to the parents of children and also informed the detailed methods and precautions. This study was reviewed and approved by the Institutional Review Board of the Second Affiliated Hospital of Dalian Medical University (Dalian, China) and adhered to the tenets of the Declaration of Helsinki. In addition, consent was obtained from the parents of patients. Explanation was provided to children using an easy-to-understand method before obtaining informed consent.

### Inclusion and exclusion criteria

The inclusion criteria were as follows: cycloplegic spherical equivalent refraction (SE) at least − 1.00 diopters (D) and diopter of spherical within − 1.00 to − 6.00 DS and myopic astigmatism ≤  − 1.00 DC and less than or equal to half the spherical diopter.

The exclusion criteria were as follows: (1) wearing contact lenses within 3 days at the start of examination, (2) children with ocular disorders such as glaucoma, cataract, keratopathy, strabismus, and amblyopia, and systemic disorders such as cardiac and respiratory illnesses, (3) intraocular pressure > 21 mm Hg and difference between the eyes > 8 mm Hg, (4) use of anticholinergic and cholinergic drugs that affect the evaluation of efficacy, such as atropine, pirenzepine, and pilocarpine within the past 1 month, (5) use of other therapies that may affect the evaluation of efficacy within the past 3 months, such as wearing OK lenses and therapy of traditional Chinese medicine, (6) low birth weight (≤ 1500 g), and (7) history of hypersensitivity to atropine or anticholinergic drugs.

### Ophthalmic examinations

We applied 1% atropine eye gel for cycloplegic autorefraction after 3 days in children aged ≤ 10 years. The compound tropicamide was applied three times with an interval of 10 min to children aged > 10 years, and we waited for 20 min for cycloplegic autorefraction.

Subjects were prescribed 0.01% atropine eye drops to be applied once per night before bedtime in both eyes in both the SA and OKA groups. Subjects were required to wear OK lenses for at least 8 h every night in the OKA and OK groups. Additionally, OK lenses were worn 10 min after the use of 0.01% atropine in the OKA group. Spectacles were rematched when the SE dropped by 0.5 D and the vision of wearing glasses decreased. OK lenses were rematched when the naked vision during the day reached < 0.5. Further, 0.01% atropine is produced by Shenyang Xingqi pharmaceutical company (Shenyang, China). There were no preservatives and the PH value between 3.5 and 4.0 in 0.01% atropine.

When the central corneal thickness stabilized after the first 1–2 months of OK treatment, the axial length measurement was used as the baseline value after 3 months as in the studies conducted by Kakita et al [8]. The patients ceased wearing OK lenses and were examined weekly until the corneal curvature regressed and the refractive error stabilized (difference between consecutive visits ≤ 0.25 D) during the follow-up visit [8]. In this study, SE was measured using the TOPCON (KR-800). IOP was measured using the TOPCON (CT-IP). Corneal topography was evaluated using the OPD-Scan III. AL was measured using the Biometer (LS 900). All experimental data were recorded using the average of five successive measurements for analysis.

### Statistical analysis

We applied SPSS 26.0 (IBM Corp., Armonk, NY) statistical analysis software for data analysis. The results of age, SE, AL, Schirmer’s test and TBuT results were described by mean (SD, standard deviation). Independent samples *t* tests were used for intergroup comparisons, and paired samples *t* test was used for intragroup comparisons. The Chi-square test was used to analyze gender differences between groups. *P* < 0.05 was considered statistically significant.

## Results

A total of 160 eyes from 80 subjects were included in this study. No statistically significant differences were found between the S and SA groups and between the OK and OKA groups in terms of age, gender, SE, AL, Schirmer’s test, or TBuT results when they started treatment (Table 1).

**Table 1:** Alteration of SE, AL, Schirmer’s test and TBuT in Children within S, SA, OK and OKA group after treatment

	S	SA	*P* value	OK	OKA	*P* value
	*n* = 20	*n* = 20		*n* = 20	*n* = 20	
Age, years, mean (SD)	9.7 (1.49)	9.65 (1.53)	0.917^a^	11.00 (1.17)	10.9 (1.29)	0.799^a^
Gender (M:F)	11:9	10:10	0.752^b^	10:10	9:11	0.752^b^
SE, D, mean (SD)						
Baseline	− 1.93 (0.74)	− 1.98 (0.45)	0.687^a^	− 2.75 (0.46)	− 2.85 (0.45)	0.293^a^
Change at 3 mo	− 2.26 (0.68)	− 2.09 (0.47)	0.203^a^	− 2.88 (0.42)	− 2.90 (0.42)	0.819^a^
Change at 6 mo	− 2.56 (0.65)	− 2.19 (0.45)	< 0.05^a^	− 2.98 (0.41)	− 2.96 (0.41)	0.801^a^
Change at 12 mo	− 3.17 (0.59)	− 2.34 (0.47)	< 0.05^a^	− 3.07 (0.44)	− 3.02 (0.43)	0.622^a^
Change over 3 mo	− 0.33 (0.14)	− 0.11 (0.07)	< 0.05^a^	− 0.14 (0.09)	− 0.05 (0.06)	< 0.05^a^
Change over 6 mo	− 0.67 (0.17)	− 0.19 (0.08)	< 0.05^a^	− 0.24 (0.15)	− 0.10 (0.08)	< 0.05^a^
Change over 12 mo	− 1.30 (0.44)	− 0.34 (0.16)	< 0.05^a^	− 0.33 (0.16)	− 0.15 (0.08)	< 0.05^a^
AL, mm, mean (SD)						
Baseline	24.28 (0.83)	24.17 (0.68)	0.500^a^	24.42 (0.48)	24.56 (0.39)	0.177^a^
Change at 3 mo	24.41 (0.82)	24.21 (0.69)	0.227^a^	24.49 (0.48)	24.58 (0.39)	0.385^a^
Change at 6 mo	24.62 (0.83)	24.64 (0.39)	< 0.05^a^	24.62 (0.51)	24.64 (0.39)	0.812^a^
Change at 12 mo	25.00 (0.87)	24.46 (0.69)	< 0.05^a^	24.72 (0.51)	24.68 (0.40)	0.732^a^
Change over 3 mo	0.13 (0.05)	0.03 (0.01)	< 0.05^a^	0.07 (0.03)	0.02 (0.02)	< 0.05^a^
Change over 6 mo	0.31 (0.06)	0.10 (0.04)	< 0.05^a^	0.19 (0.13)	0.08 (0.05)	< 0.05^a^
Change over 12 mo	0.72 (0.21)	0.24 (0.12)	< 0.05^a^	0.29 (0.11)	0.14 (0.08)	< 0.05^a^
Schirmer’s test, *s*, mean(SD)						
Baseline	18.10 (1.54)	18.20 (1.57)	0.775^a^	18.15 (1.46)	18.33 (1.90)	0.645^a^
Change at 3 mo	18.02 (1.49)	18.05 (1.21)	0.935^a^	18.08 (1.30)	18.18 (1.35)	0.738^a^
Change at 6 mo	17.95 (1.56)	17.98 (1.36)	0.940^a^	18.00 (1.01)	18.08 (1.20)	0.764^a^
Change at 12 mo	17.90 (1.49)	17.90 (1.73)	1.000^a^	17.90 (0.77)	17.98 (1.25)	0.748^a^
Change over 3 mo	− 0.07 (0.65)	− 0.12 (0.96)	0.787^a^	− 0.07 (0.85)	− 0.15 (1.02)	0.731^a^
Change over 6 mo	− 0.15 (1.09)	− 0.22 (1.00)	0.750^a^	− 0.15 (1.00)	− 0.25 (1.12)	0.676^a^
Change over 12 mo	− 0.20 (1.01)	− 0.30 (1.04)	0.665^a^	− 0.20 (1.32)	− 0.32 (1.60)	0.705^a^
TBuT, *s*, mean(SD)						
Baseline	12.60 (1.53)	12.65 (1.67)	0.889^a^	12.65 (1.38)	12.80 (1.52)	0.646^a^
Change at 3 mo	12.53 (1.66)	12.50 (1.51)	0.944^a^	10.73 (1.28)	10.78 (1.67)	0.881^a^
Change at 6 mo	12.48 (1.64)	12.45 (1.76)	0.948^a^	12.40 (1.59)	12.48 (1.64)	0.837^a^
Change at 12 mo	12.45 (1.63)	12.40 (1.73)	0.895^a^	12.48 (1.55)	12.55 (1.66)	0.835^a^
Change over 3 mo	− 0.07 (0.94)	− 0.15 (0.77)	0.698^a^	− 1.92 (0.69)	− 2.22 (0.83)	0.084^a^
Change over 6 mo	− 0.12 (0.91)	− 0.20 (0.91)	0.714^a^	− 0.25 (0.63)	− 0.32 (0.79)	0.642^a^
Change over 12 mo	− 0.15 (0.66)	− 0.25 (0.84)	0.556^a^	− 0.17 (0.67)	− 0.25 (0.87)	0.668^a^

*SE* spherical equivalent refraction, *AL* axial length, *D* dioptre, *TBuT* tear film break-up time, *M:F* male:female, *SD* standard deviation

^a^ Independent samples *t *test

^b^ Chi-square test

The increases in SE were − 0.33 ± 0.14, − 0.67 ± 0.17, and − 1.30 ± 0.44 D, respectively, in the S group and − 0.11 ± 0.07, − 0.19 ± 0.08, and − 0.34 ± 0.16 D, respectively, in the SA group (*P* < 0.05, independent samples *t* test) over 3, 6, and 12 months. The increases in SE were − 0.14 ± 0.09, − 0.24 ± 0.15, and − 0.33 ± 0.16 D, respectively, in the OK group and − 0.05 ± 0.06, − 0.10 ± 0.08, and − 0.15 ± 0.08 D, respectively, in the OKA group (*P* < 0.05, independent samples *t*-test) over 3, 6, and 12 months (Table 1).

Regarding the increases in AL, the values were 0.13 ± 0.05, 0.31 ± 0.06, and 0.72 ± 0.21 mm, respectively, in the S group and 0.03 ± 0.01, 0.10 ± 0.04, and 0.24 ± 0.12 mm, respectively, in the SA group (*P* < 0.05, independent samples *t* test) over 3, 6, and 12 months. In the OK group, the increases in AL were 0.07 ± 0.03, 0.19 ± 0.13, and 0.29 ± 0.11 mm, respectively, and those in the OKA group were 0.02 ± 0.02, 0.08 ± 0.05, and 0.14 ± 0.08 mm, respectively (*P* < 0.05, independent samples *t* test) over 3, 6, and 12 months (Table 1).

No statistically significant differences were found in Schirmer’s test and TBuT results between the S and SA groups and also between the OK and OKA groups. However, statistically significant differences were found in TBuT results between before treatment and after treatment in the OK and OKA groups (both *P* < 0.05, paired samples *t* test), especially in the first 3 months; however, it almost recovered to pretreatment levels after 1 year. The Schirmer’s test and TBuT results showed a tendency to reduce in the SA and OKA groups even when all patients exhibited no symptoms and the data showed no statistical significance (Tables 2 and 3).

**Table 2:** Alteration of Schirmer’s test in Children within S, SA, OK and OKA group after treatment

Group	Baseline	3 months	6 months	12 months	*P* value		
					Baseline versus 3 mo	Baseline versus 6 mo	Baseline versus 12 mo
S	18.10 (1.54)	18.02 (1.49)	17.95 (1.56)	17.90 (1.49)	0.474^a^	0.393^a^	0.221^a^
SA	18.20 (1.57)	18.05 (1.21)	17.98 (1.36)	17.90 (1.73)	0.323^a^	0.163^a^	0.076^a^
OK	18.15 (1.46)	18.08 (1.30)	18.00 (1.01)	17.90 (0.82)	0.584^a^	0.349^a^	0.237^a^
OKA	18.33 (1.90)	18.18 (1.35)	18.08 (1.20)	17.98 (1.25)	0.383^a^	0.168^a^	0.185^a^

^a^Paired samples *t* test

**Table 3:** Alteration of TBuT in Children within S, SA, OK and OKA group after treatment

Group	Baseline	3 months	6 months	12 months	*P* value		
					Baseline versus 3 mo	Baseline versus 6 mo	Baseline versus 12 mo
S	12.60 (1.53)	12.53 (1.66)	12.48 (1.64)	12.45 (1.63)	0.618^a^	0.391^a^	0.160^a^
SA	12.65 (1.67)	12.50 (1.51)	12.45 (1.76)	12.40 (1.73)	0.225^a^	0.173^a^	0.067^a^
OK	12.65 (1.38)	10.73 (1.28)	12.40 (1.59)	12.48 (1.55)	> 0.05^a^	0.016^a^	0.109^a^
OKA	12.80 (1.52)	10.78 (1.67)	12.48 (1.64)	12.55 (1.66)	> 0.05^a^	0.014^a^	0.077^a^

^a^Paired samples *t* test

## Discussion

With the increase in academic stress and the decrease in time spent in outdoor activities, the problem of myopia is increasing in terms of both incidence and severity in children. In addition, children-onset myopia is associated with the development of high myopia, which could result in several pathological complications such as cataracts, glaucoma, macular degeneration, retinal detachment, and even blindness [2]. Myopia has been considered as the sixth most common blindness cause, after ocular diseases such as cataracts, glaucoma, diabetic retinopathy and macular degeneration [9]. Therefore, we must take action to slow the development of myopia.

Juvenile myopia progression is mediated by both genetic and environmental factors. The cause and mechanism of the onset and progression of myopia are still not well understood. To explain the increase in the onset of myopia in school-aged children, several theories were proposed, including fewer outdoor activities, more near distance work, bad reading and writing habits, lighting, nutrition, and sleep [10, 11]. Meanwhile, several studies demonstrated that the risk of developing myopia in adolescents is significantly related to their parents because children with highly myopic parents were found to exhibit a rapid myopia progression rate [12].

Atropine and OK lenses are the commonly used methods to control myopia. Atropine is a nonselective mAChR antagonist; however, how atropine reduces the progression of myopia is unclear, although several possible mechanisms exist. A previous animal study demonstrated that atropine appears to block the M1 and M4 receptors in the retina and sclera that restrain axial elongation and reduce myopia progression by affecting the remodeling of sclera and reducing vitreous chamber growth [13]. Atropine also indirectly acts on the retina, causing retinal pigment epithelial cells or melanocytes to produce new chemicals (such as dopamine) and then acts on the sclera, which in turn inhibits thinning or stretching of the sclera to retard myopia progression [14]. Furthermore, atropine increases ultraviolet exposure in some extent due to mydriasis after using atropine may increase collagen cross-linking within the sclera, thereby limiting scleral extension [15]. Myopia may also be associated with increased ocular chronic inflammation, which may be reduced by atropine [16]. Another commonly accepted mechanism of atropine action is that it relieves nearwork-induced transient myopia (NITM). NITM is a transient myopia drift of near work for a period of time and a crucial factor related to the development of permanent myopia (PM) [17]. Currently, NITM is a complex problem that has not been well understood. Recent study has shown that 0.01% atropine have no clinical effect on corneal and lens; therefore it has almost no side effect. The effects of atropine mainly act on reducing AL elongation [18]. One study [19] reported that adverse reactions were not significantly related to 0.01% atropine.

OK is another important optical method used to control myopia progression, which is a technique that uses reverse-geometry-designed hard oxygen-permeable contact lens that is worn while sleeping to reshape the cornea momentarily to correct refractive error and improve naked vision during the day [20]. Currently, several studies show the efficacy of OK lenses in slowing the progression of myopia and axial elongation in children [21, 22]; however, the results vary greatly among individuals.

In this study, an evidently better SE control over 12 months was observed in the SA and OKA groups compared with the S and OK groups. The AL over 12 months was evidently restrained in the SA and OKA groups compared to that in the S and OK groups. Hence, 0.01% atropine can more effectively control myopia progression and axial elongation even when combined with spectacles or OK lenses.

The TBuT results were reduced after treatment with OK lenses irrespective of receiving 0.01% atropine. This is because OK lenses may influence the quality and stability of tear film. The mechanism underlying dry eye symptoms caused by wearing OK lenses remains unclear; however, several clinical studies confirmed that wearing corneal contact lenses for a long time will exhibit an effect on the amount and quality of tear secretion and dynamic distribution of tear film [23, 24]. The direct contact between OK lenses and tear film at night exhibits an effect on the physiological environment of the surface epithelium and tear, which destroys the connection between tear film and microvilli of epithelial cells, resulting in normal secretion of tear but difficult to form a stable tear film [25]. In this study, the TBuT results decreased more obviously in the first 3 months and then increased after 3 months, and it almost recovered to pretreatment levels after 1 year. All the enrolled patients exhibited no dry eye symptoms, and the experimental data showed no statistical significance during the follow-up; however, the Schirmer’s test and TBuT results showed a tendency to slightly reduce after treatment with 0.01% atropine. The possible reason is the inhibitory effect of atropine on the secretion of glands. However, the specific mechanism and the long-term effect of atropine on lacrimal secretion need to be explored.

In the current clinical treatment, questions still remain about which children would best benefit from treatment (e.g., in terms of age, level of myopia, rate of progression, and family risk factors), when atropine should be started and stopped, and for how long it should be used. Furthermore, we did not take into consideration the environmental factors such as near work time and outdoor activity time and the pupil diameters of the patients during the follow-up visits. In this study, the number of children was limited and the follow-up time was only 1 year. Therefore, further long-term follow-up studies are needed. We intend to continue to enroll additional subjects and conduct follow-up for a longer period of time.

## Conclusion

This study provides clinical evidence indicating that 0.01% atropine can effectively control myopia progression and axial elongation regardless of combined treatment with spectacles or OK lenses. The TBuT reduced after treatment with OK lenses, especially in the first 3 months irrespective of receiving 0.01% atropine. However, it almost recovered to the pretreatment levels after 1 year. Researchers also found that Schirmer and TBuT results showed a tendency to reduce after treatment with 0.01% atropine, even when all patients exhibited no symptoms and data not showing statistical significance.

## Data Availability

All data and material are available from supplementary material.
